# Risk Prediction Models for Hospital Readmission After Percutaneous Coronary Intervention: A Systematic Review and Meta-Analysis

**DOI:** 10.31083/RCM39409

**Published:** 2025-09-12

**Authors:** Yijun Mao, Hui Fan, Wenjing He, Xueqian Ouyang, Xiaojuan Wang, Erqing Li

**Affiliations:** ^1^Department of Nursing, Xianyang Central Hospital, 712000 Xianyang, Shaanxi, China; ^2^Interventional Operating Room, Xianyang Central Hospital, 712000 Xianyang, Shaanxi, China

**Keywords:** coronary heart disease, PCI, readmission, prediction model, systematic review, meta-analysis

## Abstract

**Background:**

To rigorously evaluate the methodological quality and predictive performance of risk models for hospital readmission following percutaneous coronary intervention (PCI), as well as identify key predictive factors, and evaluate potential biases along with the clinical suitability of these models.

**Method:**

An extensive search was performed across multiple databases, including PubMed, Web of Science, The Cochrane Library, Embase, Cumulative Index to Nursing and Allied Health Literature (CINAHL), China National Knowledge Infrastructure (CNKI), Wanfang Database, China Science and Technology Journal Database (VIP), and SinoMed, to identify studies on risk prediction models for hospital readmission following PCI. This search encompassed all available records from the establishment of these databases up to November 1, 2024. The screening procedure was conducted by two independent researchers, who also gathered the relevant data.

**Results:**

A total of 10 studies were incorporated, encompassing 18 models designed to predict readmission. The sample sizes across these models ranged significantly, from those containing as few as 247 participants to samples with as many as 388,078 participants. The reported incidence of readmission varied between 0.70% and 31.44%. Frequently identified predictor variables (occurring in at least four studies) included age, concurrent heart failure, diabetes, chronic lung disease, three-vessel disease, and gender. Nine models provided the area under the receiver operating characteristic (AUROC) curve, with values ranging from 0.660 to 0.899, while calibration metrics were provided in six studies. Internal validation was performed in eight studies, while one study incorporated both an internal and external validation. Eight studies were assessed and found to possess a high risk of bias, largely related to deficiencies in data analysis. The combined AUROC curve for the nine validated models was 0.80 (95% confidence interval (CI): 0.74–0.85), suggesting moderate discrimination ability.

**Conclusion:**

Although existing risk prediction models for hospital readmission following PCI demonstrate a moderate level of predictive discrimination, most of the included studies were found to have a high risk of bias according to the Prediction model Risk Of Bias ASsessment Tool (PROBAST). Therefore, future studies should aim to develop more robust models using larger sample sizes, rigorous methodologies, and multicenter external validation.

**The PROSPERO Registration:**

CRD42024616342. https://www.crd.york.ac.uk/PROSPERO/view/CRD42024616342.

## 1. Introduction

Although percutaneous coronary intervention (PCI) enhances survival and quality of life for patients with coronary heart disease, early post-discharge readmissions remain common and are often preventable [1, 2]. Consequently, understanding and predicting these readmissions is essential for optimizing patient care and healthcare resource allocation [3, 4].

Cardiovascular disease remains a significant global health challenge, with coronary heart disease representing the primary cause of mortality among individuals with cardiovascular conditions [5]. PCI is currently the most widely utilized procedure for coronary revascularization, effectively restoring coronary blood flow, reducing myocardial infarction severity, and enhancing long-term patient prognosis [6]. Although PCI techniques have advanced considerably and offer clear clinical benefits for appropriately selected patients, post-procedural complications remain a significant challenge, frequently leading to hospital readmissions that exacerbate disease burden, psychological distress, and healthcare expenditures [7, 8]. Early readmission after PCI is regarded as a frequent and costly adverse event [9]. In the United States, approximately 20% of patients undergoing PCI experience unplanned readmissions annually, significantly contributing to healthcare expenditures [10]. When both planned and unplanned readmissions are considered, the rate rises to approximately 25–30% [11], with unplanned readmissions alone amounting for an estimated $26 billion in potentially avoidable costs [12]. Furthermore, 75% of these readmissions are considered preventable [13, 14, 15], with the highest-risk period being the first seven days post-discharge and most occurring within 30 days [10]. Reported 30-day readmission rates range from 4.7% to 22.0%, though these figures may underestimate true rates due to external hospitals readmissions [16, 17].

Readmission risk prediction models for patients undergoing PCI incorporate a range of predictive factors, however, current approaches face several critical limitations. First, existing models often demonstrate inconsistent performance across diverse populations and exhibit poor generalizability beyond their derivation cohorts [18]. The most widely utilized general model, the length of stay, acuity of admission, comorbidity of the patient, and number of emergency department visits (LACE) index [19, 20, 21], although validated in broad populations, fails to incorporate PCI-specific risk factors and demonstrates variable predictive accuracy for cardiac-related readmissions [22]. Second, many models suffer from methodological weaknesses, including inadequate handling of missing data, suboptimal variable selection, absence of internal validation, and insufficient calibration assessment [23, 24, 25]. Few have undergone rigorous external validation, and those that have typically exhibit significantly reduced performance [25]. Furthermore, most studies do not evaluate both discrimination and calibration, thereby limiting clinical interpretability [26]. Third, clinical applicability remains limited. Many models rely on variables unavailable at the point of care or require complex calculations impractical in routine clinical settings [27]. Few have been assessed for implementation feasibility or impact on clinical outcomes [28]. Finally, the rapid advancement of PCI techniques and adjunctive therapies has rendered several older models obsolete, as they fail to reflect contemporary clinical practice [29].

Despite the proliferation of PCI readmission risk models, no comprehensive evaluation has systematically assessed their methodological quality, predictive performance, and clinical utility. Previous reviews have either narrowly focused on specific models [30] or lacked rigorous quality appraisal using contemporary tools such as Prediction model Risk Of Bias ASsessment Tool (PROBAST) [31]. Moreover, no meta-analysis has quantitatively synthesized model performance across studies. This systematic review and meta-analysis aims to address these gaps by: (1) comprehensively evaluating the methodological rigor of existing models using the PROBAST and Checklist for Critical Appraisal and Data Extraction for Systematic Reviews of Prediction Modelling Studies (CHARMS) frameworks; (2) quantitatively synthesizing their predictive performance through meta-analysis; and (3) providing evidence-based recommendations for model selection and future research. The findings are intended to assist clinicians in selecting appropriate risk prediction tools and to inform the development of more robust and clinically applicable models.

## 2. Methods

We utilized the population, intervention, comparator, outcomes, timing, and setting (PICOTS) framework to structure the clinical inquiry (**Supplementary Table 1**). The study is registered with PROSPERO under registration number CRD42024616342.

### 2.1 Search Strategy

We performed a comprehensive search across multiple databases, including PubMed, Web of Science, The Cochrane Library, Embase, Cumulative Index to Nursing and Allied Health Literature (CINAHL), China National Knowledge Infrastructure (CNKI), Wanfang Database, China Science and Technology Journal Database (VIP), and SinoMed, covering all records from their establishment up to November 1, 2024. The search strategy targeted three primary concepts: PCI, readmission, and prediction. The core Boolean logic used was: “(PCI OR percutaneous coronary intervention) AND (readmission OR rehospitalization) AND (prediction OR prognostic OR risk model)”. Detailed, database-specific search strategies are provided in **Supplementary Table 2**.

### 2.2 Inclusion and Exclusion Criteria

The inclusion criteria were: (1) research involving individuals who underwent PCI; (2) observational cohort or case-control studies; (3) readmission post-PCI as the reported outcome; and (4) inclusion of a predictive model. Exclusion criteria encompassed: (1) research focusing solely on risk factors for readmission without developing risk prediction models; (2) studies lacking accessible full texts; (3) non-peer-reviewed materials, such as conference abstracts and agency reports; (4) redundant or overlapping publications; and (5) studies published in languages other than English or Chinese.

### 2.3 Study Selection and Data Extraction

Two reviewers (MY and HW) independently screened articles according to the predefined inclusion criteria, with discrepancies resolved by a third reviewer (FH).

Data extraction was conducted using the CHARMS framework (see **Supplementary Table 3**). The extracted data from the selected studies were classified into four categories: (1) General study information, including the first author, year of publication, study design, data source, study period, and outcome definition; (2) Basic model information, encompassing sample size, outcome event rate, events per variable (EPV), model development method, variable selection method, handling of missing data, and processing of continuous variables; (3) Model performance, covering discrimination, calibration, type of validation, and formats for presenting the risk prediction models; and (4) Predictors, detailing the number of candidate variables and final predictors.

### 2.4 Quality Assessment

The risk of bias and applicability of the included studies were assessed using the PROBAST. The risk of bias assessment encompassed all four PROBAST domains: participant selection, predictors, outcome, and analysis. Results were stratified by domain, and the risk of bias was visualized using the robvis tool.

### 2.5 Data Synthesis and Statistic Analysis

A meta-analysis was conducted to assess the discriminative performance of readmission prediction model, as measured by the area under the receiver operating characteristic curve (AUC). The analysis followed a structured process. First, to stabilize variance, AUC values were transformed to the logit scale using the formula: $l\,o\,g\,i\,t\,\left(A\,U\,C\right)=\ln\left(\frac{A\,U\,C}{1-A\,U\,C}\right)$. The standard error was calculated using the expression: $S\,E_{l\,o\,g\,i\,t\,\left(A\,U\,C\right)}=\sqrt{\frac{A\,U\,C\,\left(1-A\,U\,C\right)}{n\cdot \phi^{2}}}$, where ϕ denotes the probability density function at the optimal cutoff point. Effect sizes were pooled using the inverse-variance weighting method. Heterogeneity was evaluated using the Q-test and the I^2^ statistic. Substantial heterogeneity was defined as I^2^ > 50% and Q-test *p* ≤ 0.1, warranting the use of a random-effects model. In contrast, low heterogeneity (I^2^ ≤ 50% and Q-test *p* > 0.1) supported the use of a fixed-effects model. For analyses with significant heterogeneity (I^2^ > 50%), subgroup analyses were conducted to explore potential sources of variability, stratified by publication date, study region, type of readmission, and modeling methods. Funnel plot asymmetry was assessed both visually and statistically using Egger’s linear regression test, with *p* < 0.05 considered indicative of potential publication bias. All analyses were performed in R (version 4.4.1; R Foundation for Statistical Computing, Vienna, Austria) using the meta and metafor packages.

## 3. Results

### 3.1 Study Selection

**Fig. 1.**
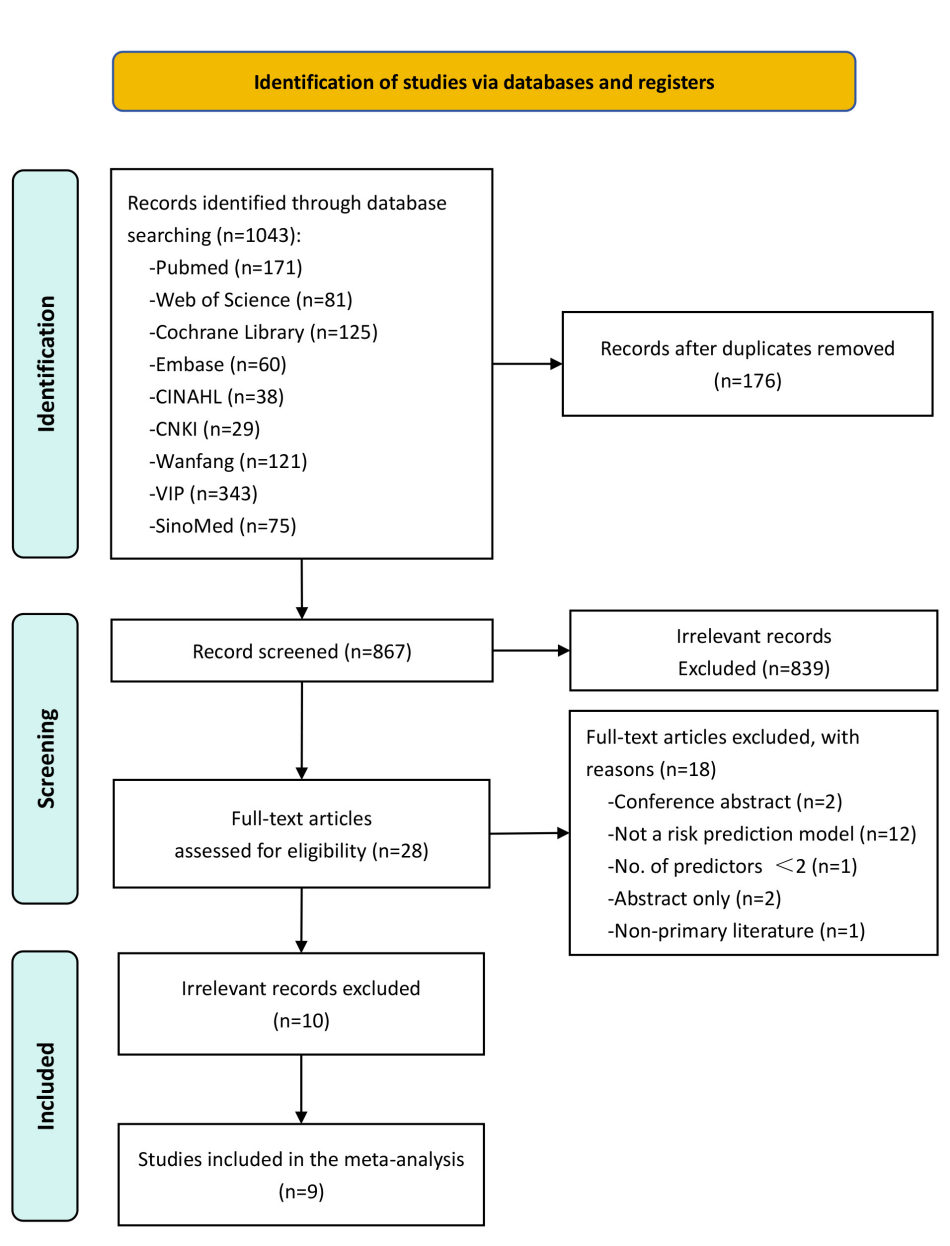
**Flowchart of the study selection process**. CINAHL, Cumulative Index to Nursing and Allied Health Literature; CNKI, China National Knowledge Infrastructure; VIP, China Science and Technology Journal Database.

From the database searches, 1043 records were retrieved, of which 176 duplicates were excluded. Following the screening of 867 articles, 839 were excluded as irrelevant. A further 28 articles were eliminated for specific reasons: conference abstracts (n = 2), absence of a risk prediction model (n = 12), fewer than two predictors (n = 1), abstract only (n = 2), and non-primary literature (n = 1). In the end, 10 studies met the inclusion criteria, presenting a combined total of 18 prediction models for hospital readmission following PCI (Fig. 1).

### 3.2 Study Characteristics

The fundamental characteristics of the included studies are summarized in Table 1 (Ref. [17, 32, 33, 34, 35, 36, 37, 38, 39, 40]). These studies were published from 2013 and 2024, with six conducted in China and four in the United States. Among these, eight studies adopted a retrospective cohort design, while two followed a prospective cohort design. Seven studies recruited patients from a single center, while the remaining three reported results from two or more centers. Regarding study populations, nine investigations examined individuals with coronary heart disease, including two that specifically concentrated on elderly individuals aged 60 and older. One study focused on participants with acute coronary syndrome and type 2 diabetes mellitus. Sample sizes varied from 247 to 388,078 participants, with the mean or median age of participants spanning 59 to 73 years. The outcomes of interest included heart-related readmissions (n = 6), with subcategories such as myocardial infarction-related readmissions (n = 2), major adverse cardiac events (MACE)-related readmissions (n = 2), and congestive heart failure (CHF)-related readmissions (n = 1). Four studies investigated all-cause readmissions. Follow-up periods for readmission varied, with five studies evaluating 30-day readmissions and another five assessing 1-year readmissions. Reported readmission rates varied widely: 30-day all-cause readmission rates spanned from 7.94% to 11.36%, while 1-year heart-related readmission rates ranged from 16.93% to 31.44%.

**Table 1.** **Basic characteristics of the included studies**.

First author (year)	Country	Study design	Research object	Data source location (Study period)	Outcome definition		Readmission cases/sample size (%)		Population characteristics	
					Type of readmission	Length of time	Training set	Validation set	Non-readmission	Readmission
Wasfy 2013 [39]	US	Retrospective study	Patients >18 years old who underwent PCI	Massachusetts Data Analysis Center (2005–2008)	all-cause readmission	30 d	2500/24,040 (10.40%)	-/12,020	Age: 64.3 ± 12.4 Male: 70.5%	Age: 68.1 ± 13.1 Male: 59.9%
Minges 2017 [37]	US	Prospective study	Patients >65 years old who underwent PCI	The National Cardiovascular Data Registry (NCDR) CathPCI Registry (2007–2009)	all-cause readmission	30 d	22,027/193,899 (11.36%)	22,039/194,179 (11.35%)	Age: 72.5 Male: 59.1%	
Fanari 2017 [40]	US	Retrospective study	Patients >18 years old who underwent PCI	Christiana Care Health System (2010–2015)	all-cause readmission	30 d	318/3739 (8.50%)	-/2978	Age: 64.2 ± 12.1 Male: 68.7%	
Zack 2019 [35]	US	Prospective study	Patients >18 years old who underwent PCI	Mayo Clinic PCI Registry (2004–2013)	CHF readmission	30 d	82/11,709 (0.70%)	-	Age: 66.9 ± 12.2 Male: 71.5%	
Xu 2022 [17]	China	Retrospective study	Patients >18 years old who underwent PCI	Department of Cardiology at the Second Affiliated Hospital of Nanchang University (2020)	all-cause readmission	30 d	107/1348 (7.94%)	-	Age: 66.39 ± 11.12 Male: 73.3%	
Zhang 2022 [36]	China	Retrospective study	Patients with AMI >18 years old who underwent PCI	Cardiovascular department of the Second People’s Hospital of Huaian (2018–2020)	AMI-related readmission	1 y	42/247 (17.00%)	-	Age: 59.2 ± 4.6 Male: 65.6%	
Zhang 2022 [33]	China	Retrospective study	Patients with CHD >60 years old who underwent PCI	Cardiovascular department of the Third Hospital of Xingtai (2017–2019)	Heart-related readmission	1 y	133/423 (31.44%)	108/-	Age: 71.21 ± 3.27 Male: 74.9%	
Yao 2022 [34]	China	Retrospective study	STEMI patients >18 years old who underwent PCI	Cardiovascular department of the First Hospital of Jilin University (2016)	MACE-related readmission	1 y	70/526 (13.31%)	-	Age: 59 ± 11 Male: 71.9%	
Zhang 2024 [38]	China	Retrospective study	Patients with ACS and DM >18 years old who underwent PCI	Cardiovascular department of Xijing Hospital, Air Force Medical University (2019–2022)	MACE-related readmission	1 y	236/1390 (17.00%)	122/597 (20.40%)	Age: 64 (57, 70) Male: 75.4%; 72.0%	
Liu 2024 [32]	China	Retrospective study	NSTEMI patients >18 years old who underwent PCI	Cardiovascular department of the Affiliated Hospital of North Sichuan Medical University (2014–2022)	myocardial infarction-related readmission	1 y	162/957 (16.93%)	64/406 (15.76%)	Age: 64.05 ± 11.39 Male: 73.2%	Age: 68.74 ± 10.55 Male: 67.7%

AMI, acute myocardial infarction; CHD, coronary heart disease; STEMI, ST-segment elevation myocardial infarction; ACS, acute coronary syndrome; DM, diabetes mellitus; NSTEMI, non-ST-segment elevation myocardial infarction; CHF, congestive heart failure; MACE, major adverse cardiac events.

**Fig. 2.**
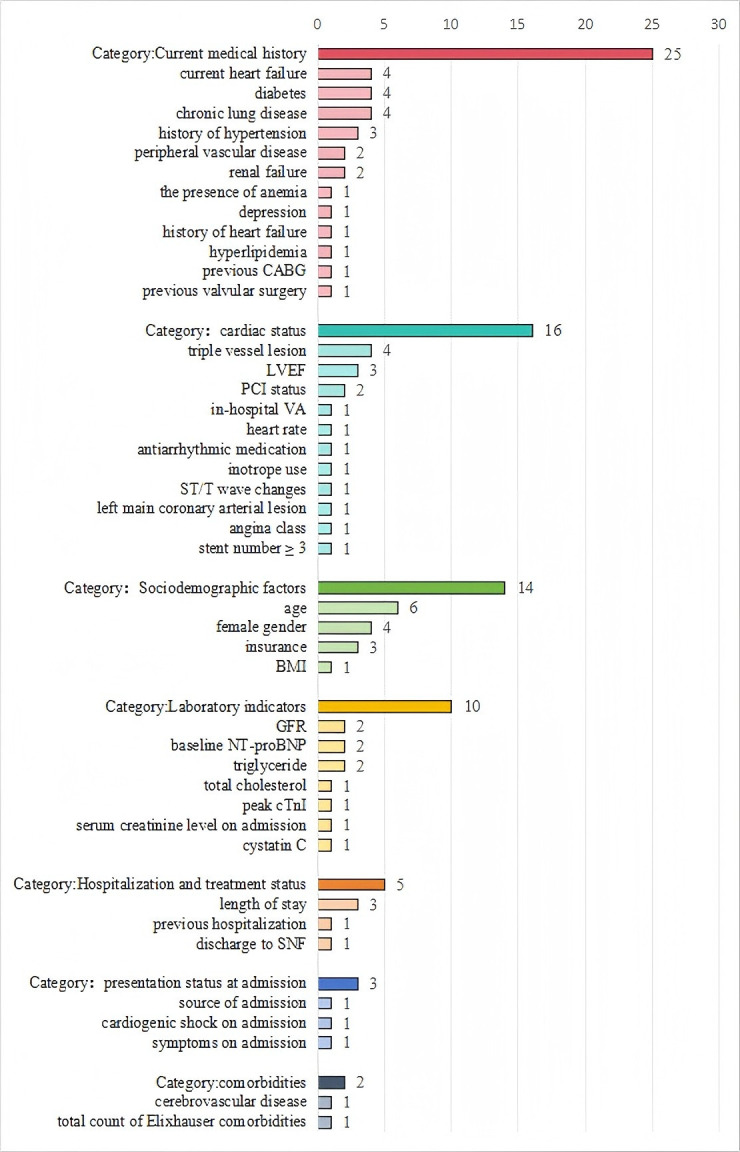
**Frequency of predictor variables used in included studies**. The numerical values adjacent to each predictor indicate the number of studies (out of 10 total included studies) that incorporated the variable into their prediction models. For example, “4” next to “diabetes” means that diabetes was included as a predictor in 4 studies. CABG, coronary artery bypass grafting; LVEF, left ventricular ejection fraction; VA, ventricular arrhythmia; ST/T, ST-segment/T-wave; BMI, body mass index; GFR, glomerular filtration rate; NT-proBNP, N-terminal pro B-type natriuretic peptide; SNF, skilled nursing facility; PCI, percutaneous coronary intervention.

The model information is detailed in Table 2 (Ref. [17, 32, 33, 34, 35, 36, 37, 38, 39, 40]) (The complete version is provided in **Supplementary Table 4**). Logistic regression was the primary method for model development, utilized in all included studies. Additionally, machine learning methods were applied in 2 of the 10 included studies. Specifically, 2 studies employed random forest (RF), while 1 study each used decision tree (DT), support vector machine (SVM), eXtreme Gradient Boosting (XGBoost), and Adaptive Boosting (AdaBoost). Fig. 2 summarizes all predictors included in the final models. Age was the most frequently used predictor, appearing in seven models. Other common predictors included heart failure, diabetes, chronic lung disease, and triple vessel lesion, each used in four models.

**Table 2.** **Characteristics of PCI readmission risk prediction models**.

First author (year)	EPV	Continuous variable processing method	Variable selection	Model development method	Calibration method	Validation method	No. of final predictors	Optimal model performance	Model presentation
Wasfy 2013 [39]	74	Continuous variables	Backwards elimination	-	Hosmer-Lemeshow test	Split random	10	A: 0.690	Risk score, web-based calculator
								B: 0.670	
Minges 2017 [37]	2203	Categorical variables	Stepwise selection (LR model)	-	-	Split random	14	A: 0.670	Risk score
								B: 0.660	
Fanari 2017 [40]	12	Continuous variables	combination of forward selection and backward elimination	LR	Calibration plot	Bootstrapping & temporal validation	11	A: 0.752 (0.724, 0.781)	-
Zack 2019 [35]	0.2	Continuous variables	-	LR, RF	-	Cross-validation	3	0.899 (0.890, 0.910)	-
Xu 2022 [17]	3	Categorical variables	Univariate analysis and LASSO regression	LR	Hosmer-Lemeshow test	Bootstrapping	7	A: 0.770 (0.746, 0.792)	Nomogram model
Zhang 2022 [36]	1	Continuous variables	Univariate analysis	LR	Hosmer-Lemeshow test	-	4	A: 0.843	Nomogram model
Zhang 2022 [33]	7	Continuous variables	Univariate analysis	LR	-	External temporal validation	9	A: 0.828	Nomogram model
								B: 0.805	
Yao 2022 [34]	2	Continuous variables	Univariate analysis; clinical relevance, and the number of events available	LR	Hosmer-Lemeshow test	Internal validation	6	A: 0.723 (0.665, 0.780)	Nomogram model
Zhang 2024 [38]	16	Continuous variables	LASSO regression; RF; best subset selection	LR	-	Bootstrapping	7	A: 0.787 (0.760, 0.815)	Nomogram model
Liu 2024 [32]	2	Continuous variables	Univariate and multivariable logistic regression model; LASSO regression; RF	LR, DT, RF, SVM, XGBoost, AdaBoost	Calibration curve	Internal temporal validation	7	A: 0.749 (0.681, 0.817)	-

AdaBoost, adaptive boosting; DT, decision tree; EPV, events per variable; LASSO, least absolute shrinkage and selection operator regression; LR, logistic regression; RF, random forest; SVM, support vector machine; XGBoost, extreme gradient boosting; AUC, area under the curve. A, development cohort; B, validation cohort. We considered AUC = 0.5–0.7 as poor discrimination, 0.7–0.8 as moderate discrimination, 0.8–0.9 as good discrimination, and 0.9–1.0 as excellent discrimination.

Model discrimination was reported in nine studies, with C-statistic values varying between 0.660 and 0.899. Calibration was assessed in seven studies, most commonly using the Hosmer-Lemeshow test.

### 3.3 Models Validation

Among the included studies, eight conducted internal validation, with three utilizing bootstrapping, two employing random splitting, one applying cross-validation, and one adopting temporal validation. One study did not specify the internal validation method used. Additionally, one study performed both internal and external validation. The complete set of model features is presented in **Supplementary Table 4**.

### 3.4 Results of Quality Assessment

**Fig. 3.**
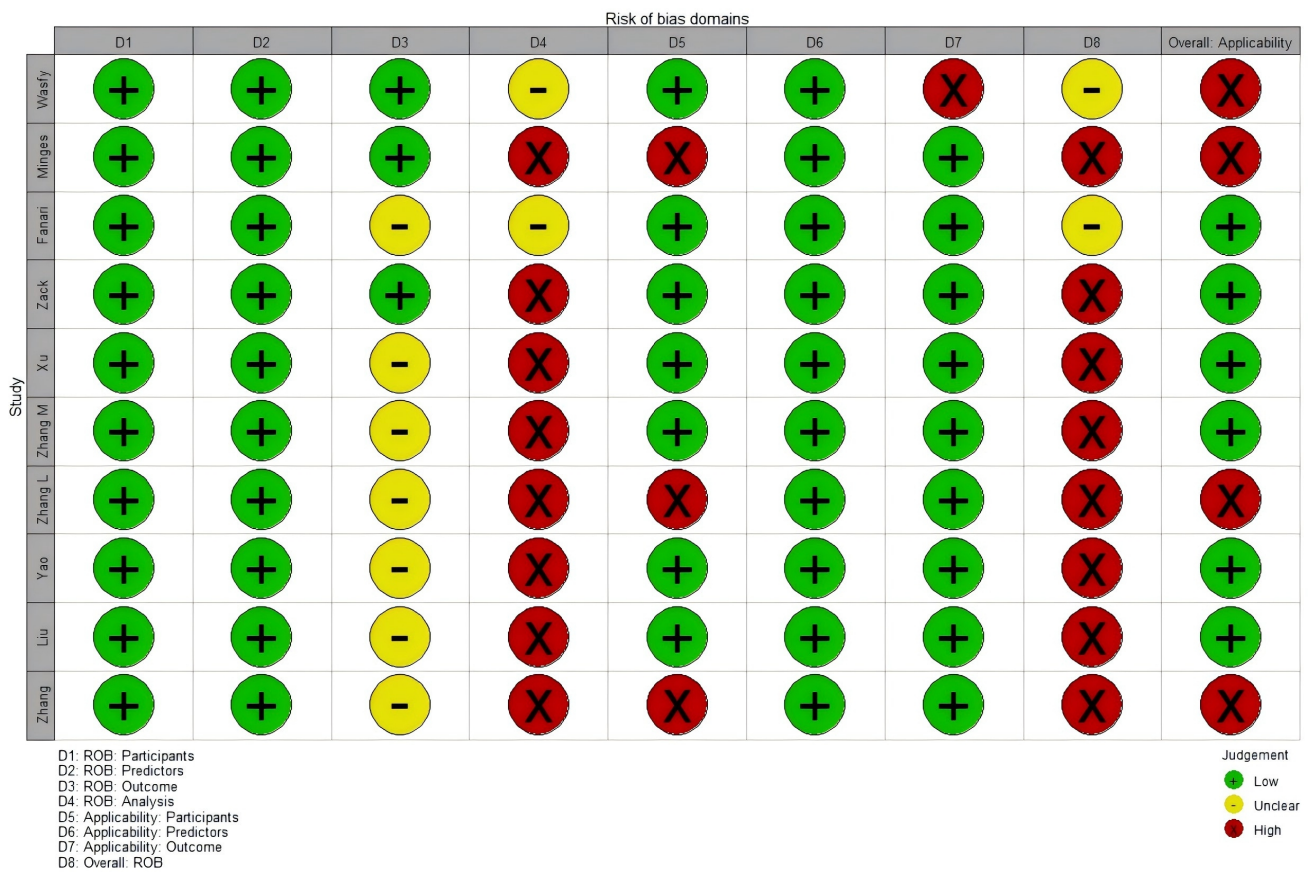
**ROB and applicability assessment of models by using PROBAST**.

Table 3 (Ref. [17, 32, 33, 34, 35, 36, 37, 38, 39, 40]) and Fig. 3 summarize the risk of bias and applicability assessments for the included studies (The bias risk assessment criteria and procedures are detailed in **Supplementary Table 5**). Eight studies were deemed to have a high risk of bias, while four were identified to carry a high risk of applicability.

**Table 3.** **Risk of bias appraisal results of eligible articles adapted from PROBAST**.

Study	Study type	ROB				Applicability			Overall	
		Participants	Predictors	Outcome	Analysis	Participants	Predictors	Outcome	ROB	Applicability
Wasfy 2013 [39]	B	+	+	+	?	+	+	–	?	–
Minges 2017 [37]	B	+	+	+	–	–	+	+	–	–
Fanari 2017 [40]	B	+	+	?	?	+	+	+	?	+
Zack 2019 [35]	B	+	+	+	–	+	+	+	–	+
Xu 2022 [17]	B	+	+	?	–	+	+	+	–	+
Zhang M 2022 [36]	A	+	+	?	–	+	+	+	–	+
Zhang L 2022 [33]	B	+	+	?	–	–	+	+	–	–
Yao 2022 [34]	A	+	+	?	–	+	+	+	–	+
Liu 2024 [32]	B	+	+	?	–	+	+	+	–	+
Zhang 2024 [38]	B	+	+	?	–	–	+	+	–	–

PROBAST, Prediction model Risk Of Bias ASsessment Tool; ROB, risk of bias. A indicates “development only”; B indicates “development and validation in the same publication”. + indicates low ROB/low concern regarding applicability; – indicates high ROB/high concern regarding applicability; ? indicates unclear ROB/unclear concern regarding applicability.

In the “**participants**” domain, all ten studies were classified as having a low risk of bias, as they either employed prospective cohort designs or retrospective cohorts that included all or consecutive PCI patients over a defined period. Within the “**predictor**” domain, all ten studies exhibited a low risk of bias. These were predominantly single-center studies, where commonly used predictors had standardized measurement protocols, and fixed variables like age and sex were inherently less susceptible to measurement bias. In the “**outcome**” domain, seven studies were also classified to have an unclear risk of bias because they failed to report whether outcomes and predictors were assesses independently (i.e., blind assessment) [32, 33].

In the “**analysis**” domain, eight model development studies were judged to have a high risk of bias. Specific issues included the following:

∙ Six studies had inadequate sample sizes, failing to meet the criterion of more than 10 EPV [17, 32, 33, 34, 35, 36].

∙ One study categorized continuous variables, potentially resulting in a loss of information [37].

∙ Three studies relied on univariate analysis for variable selection [33, 34, 36].

∙ Four studies did not comprehensively evaluated the predictive performance of their models [33, 35, 37, 38].

∙ Three study failed to address model overfitting, underfitting, or optimism in performance assessment [36, 37, 39].

∙ Five studies did not report the coefficients of predictors in their multivariate regression model [32, 35, 37, 39, 40].

∙ Two studies relied exclusively on internal validation using a single random split sample [37, 39].

∙ None of the studies reported complexities inherent in the data.

Two studies conducted external validation of previously developed predictive models. One study included at least 100 participants with outcome events in the validation cohort, meeting recommended sample size criteria. The other study did not report the number of outcome events but involved a large sample size (n = 2978), likely providing sufficient statistical power. Both studies applied the same dichotomization strategies and cut-off thresholds as defined in the original models.

For applicability, in the “**patients**” domain, three studies were classified as having a high risk of applicability because they focused on elderly patients with coronary heart disease and concurrent diabetes [33, 37, 38]. In the “**outcome**” domain, one study was assessed to have a high risk of applicability because the outcomes included not only readmission but also cardiovascular and all-cause mortality [35].

### 3.5 Meta-Analysis of Validation Models Included in the Review

This study conducted a meta-analysis of relevant factors quantitatively synthesized from 10 studies, encompassing 14 predictors. Table 4 presents the primary meta-analysis results for all investigated predictors, including pooled effect sizes (odds ratios [ORs]) and heterogeneity statistics. Random- or fixed-effects models were applied based on the degree of heterogeneity. In contrast, Table 5 provides sensitivity analyses comparing fixed- and random-effects models for each predictor to assess the robustness of the findings. The meta-analysis identified the following factors as significantly influencing readmission after PCI: age, heart failure, diabetes, chronic lung disease, triple vessel lesion, female gender, hypertension, peripheral vascular disease, renal failure, glomerular filtration rate (GFR), N-terminal pro B-type natriuretic peptide (NT-proBNP), and triglycerides. Sensitivity analyses revealed no significant differences between fixed- and random-effects models, indicating that the pooled estimates were stable and robust.

**Table 4.** **Primary meta-analysis of predictors for hospital readmission after PCI: Pooled odds ratios (ORs) with 95% CIs, heterogeneity tests, and model selection**.

Predictors	No studies*	Heterogeneity test		Effects models	Meta-analysis		
		I^2^ (%)	*p*		OR (95% CI)	Z	*p*
Age >40	6	82	<0.001	random effects models	1.14 (1.06, 1.22)	3.57	<0.001
Current heart failure	4	64	0.040	random effects models	1.38 (1.25, 1.53)	6.35	<0.001
Diabetes	4	69	0.020	random effects models	1.77 (1.34, 2.33)	4.01	<0.001
Chronic lung disease	4	57	0.070	random effects models	1.49 (1.33, 1.66)	7.01	<0.001
Triple vessel lesion	4	84	<0.001	random effects models	2.51 (1.27, 4.94)	2.66	0.008
Female gender	4	59	0.060	random effects models	1.31 (1.19, 1.44)	5.70	<0.001
History of hypertension	3	47	0.150	fixed effects models	1.56 (1.20, 2.04)	3.26	0.001
LVEF <60	3	96	<0.001	random effects models	1.13 (0.95, 1.35)	1.40	0.160
Length of stay	2	62	0.110	random effects models	1.26 (0.87, 1.82)	1.22	0.220
Peripheral vascular disease	2	0	0.770	fixed effects models	1.23 (1.19, 1.28)	10.68	<0.001
Renal failure	2	0	0.520	fixed effects models	1.55 (1.41, 1.70)	9.38	<0.001
GFR <30	2	0	0.790	fixed effects models	1.74 (1.64, 1.85)	18.53	<0.001
NT-proBNP	2	0	1.000	fixed effects models	1.00 (1.00, 1.00)	2.83	0.005
Triglyceride >0.6	2	21	0.260	fixed effects models	2.46 (1.79, 3.38)	5.56	<0.001

Fixed-effects models were used if I^2^ < 50% and *p* ≥ 0.10; otherwise, random-effects models were applied. * Only studies with complete OR and 95% CI were included.

**Table 5.** **Sensitivity analysis comparing fixed- and random-effects models for predictors of readmission after PCI**.

Predictors	No studies	Fixed effects models		Random effects models	
		OR (95% CI)	*p*	OR (95% CI)	p
Age >40	6	1.06 (1.06, 1.07)	<0.001	1.14 (1.06, 1.22)	<0.001
Current heart failure	4	1.33 (1.29, 1.38)	<0.001	1.38 (1.25, 1.53)	<0.001
Diabetes	4	1.42 (1.36, 1.48)	<0.001	1.77 (1.34, 2.33)	<0.001
Chronic lung disease	4	1.49 (1.44, 1.54)	<0.001	1.49 (1.33, 1.66)	<0.001
Triple vessel lesion	4	1.82 (1.44, 2.30)	<0.001	2.51 (1.27, 4.94)	0.008
Female gender	4	1.28 (1.24, 1.32)	<0.001	1.31 (1.19, 1.44)	<0.001
History of hypertension	3	1.56 (1.20, 2.04)	0.001	1.62 (1.11, 2.36)	0.010
LVEF <60	3	1.05 (1.03, 1.06)	<0.001	1.13 (0.95, 1.35)	0.160
Length of stay	2	1.11 (1.06, 1.17)	<0.001	1.26 (0.87, 1.82)	0.220
Peripheral vascular disease	2	1.23 (1.19, 1.28)	<0.001	1.23 (1.19, 1.28)	<0.001
Renal failure	2	1.55 (1.41, 1.70)	<0.001	1.55 (1.41, 1.70)	<0.001
GFR <30	2	1.74 (1.64, 1.85)	<0.001	1.74 (1.64, 1.85)	<0.001
NT-proBNP	2	1.00 (1.00, 1.00)	0.005	1.00 (1.00, 1.00)	0.005
Triglyceride >0.6	2	2.46 (1.79, 3.38)	<0.001	2.58 (1.65, 4.03)	<0.001

Consistency between models suggests robustness of results.

**Fig. 4.**
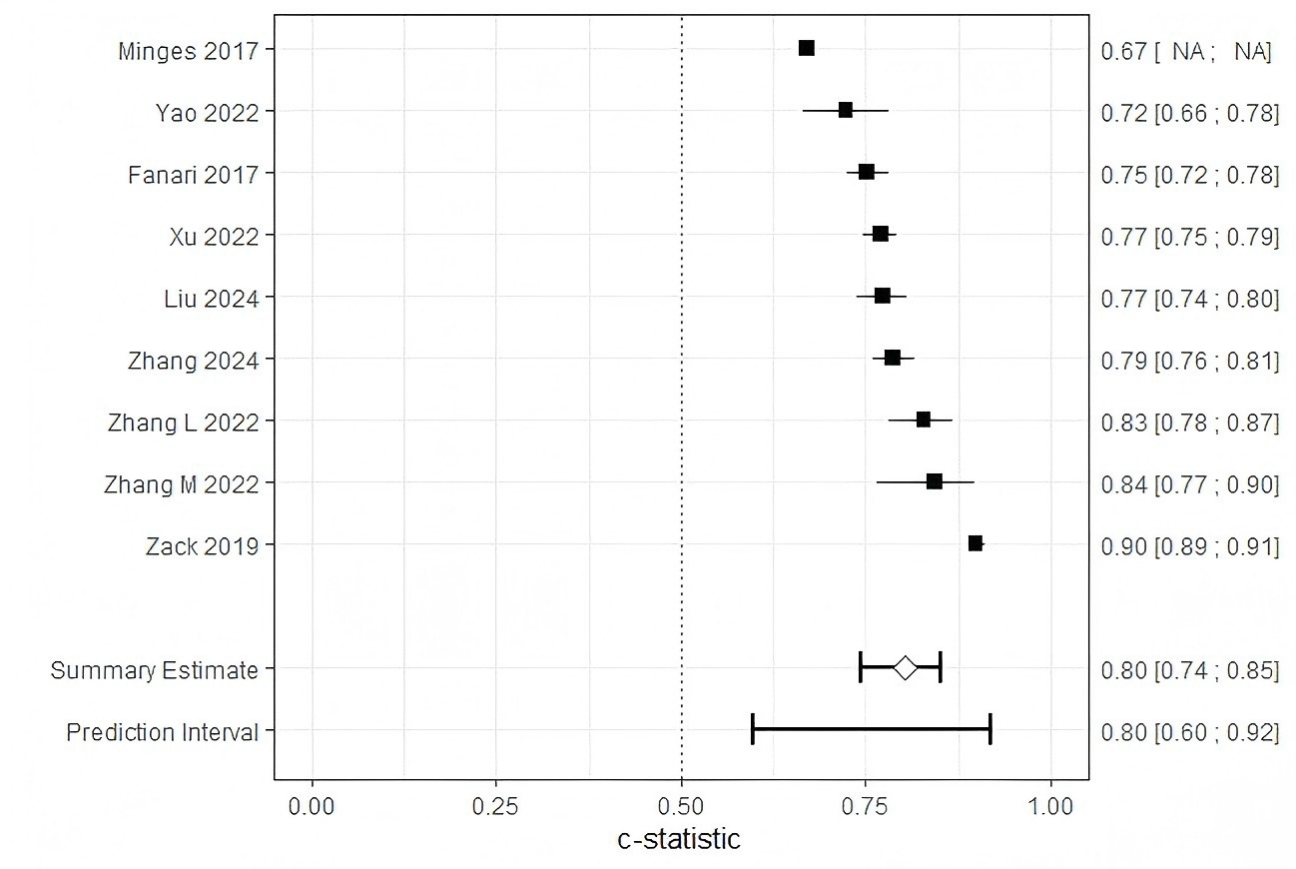
**Forest plot of the meta-analysis of pooled AUC estimates for predictive models performance**. Pooled AUC estimates should be interpreted with caution due to potential heterogeneity in outcome definitions and model development methods. NA, not available.

Of the 10 studies, one reported insufficient details on model development and was excluded, leaving 9 studies eligible for further analysis. The combined AUC for these studies was 0.80 (95% CI: 0.74–0.85) (Fig. 4). However, substantial heterogeneity was observed across studies (I^2^ = 97%, *p* < 0.001), prompting subgroup analyses to investigate potential sources of variability in readmission following PCI (see Table 6).

**Table 6.** **Results of heterogeneity analyses on readmission after PCI**.

Predictors	Subgroups	No studies	Heterogeneity test		Effects models	Meta-analysis		
			I^2^ (%)	*p*		OR (95% CI)	Z	*p*
Publication data	2013–2019	2	99	<0.001	random effects models	0.82 (0.69, 0.98)	2.18	0.030
	2020–2024	6	55	0.050	random effects models	0.78 (0.76, 0.81)	15.97	<0.001
Study region	Americas	2	99	<0.001	random effects models	0.82 (0.69, 0.98)	2.18	0.030
	Asian	6	55	0.050	random effects models	0.78 (0.76, 0.81)	15.97	<0.001
Type of readmission	All-cause readmission	2	0	0.350	fixed effects models	0.76 (0.74, 0.78)	21.80	<0.001
	MACE-related readmission	2	70	0.070	random effects models	0.76 (0.70, 0.83)	6.59	<0.001
	Heart-related readmission	4	95	<0.001	random effects models	0.83 (0.76, 0.91)	3.88	<0.001
Modeling methods	LR	7	57	0.030	random effects models	0.78 (0.76, 0.80)	18.09	<0.001
	ML methods	1	Not applicable					

ML, machine learning.

#### 3.5.1 Subgroup Analysis by Publication Year

Among studies published between 2013 and 2019 (n = 2), heterogeneity remained high (I^2^ = 99%, *p* < 0.001), with a random-effects model yielding a pooled OR of 0.82 (95% CI: 0.69–0.98; Z = 2.18; *p* = 0.030). In contrast, studies published between 2020 and 2024 (n = 6) demonstrated moderate heterogeneity (I^2^ = 55%, *p* = 0.050), with a pooled OR of 0.78 (95% CI: 0.76–0.81; Z = 15.97; *p* < 0.001) under a random-effects model. More recent studies (2020 onward) produced more consistent effect estimates with narrower confidence intervals, potentially reflecting improved methodological rigor and larger sample sizes in contemporary research.

#### 3.5.2 Subgroup Analysis by Study Region

Studies conducted in the Americas (n = 2) exhibited high heterogeneity (I^2^= 99%, *p* < 0.001), with a random-effects model yielding a pooled OR of 0.82 (95% CI: 0.69–0.98; Z = 2.18; *p* = 0.030). In contrast, studies from Asia (n = 6) demonstrated moderate heterogeneity (I^2^ = 55%, *p* = 0.050), and the random-effects model produced a pooled OR of 0.78 (95% CI: 0.76–0.81; Z = 15.97; *p* < 0.001). The more consistent effect sizes observed in Asian studies may reflect greater homogeneity in population characteristics or healthcare delivery practices.

#### 3.5.3 Subgroup Analysis by Readmission Type

Among studies examining cardiac-related readmissions (n = 4), high heterogeneity was observed (I^2^ = 95%, *p* < 0.001), with a random-effects model yielding a pooled OR of 0.83 (95% CI: 0.76–0.91; Z = 3.88; *p* < 0.001). Studies evaluating major adverse cardiac event (MACE)-related readmissions (n = 2) showed moderate heterogeneity (I^2^ = 70%, *p* = 0.070), with a pooled OR of 0.76 (95% CI: 0.70–0.83; Z = 6.59; *p* < 0.001) under a random-effects model. In contrast, studies investigating all-cause readmissions (n = 2) exhibited no heterogeneity (I^2^ = 0%, *p* = 0.350), and a fixed-effects model estimated a pooled OR of 0.76 (95% CI: 0.74–0.78; Z = 21.80; *p* < 0.001). The observed heterogeneity may be attributable to differences in the operational definitions of “cardiac-related” and “MACE” outcomes across studies.

#### 3.5.4 Subgroup Analysis by Modeling Methods

Among studies employing logistic regression (LR) models (n = 7), moderate heterogeneity was observed (I^2^ = 57%, *p* = 0.030), and a random-effects model yielded a pooled OR of 0.78 (95% CI: 0.76–0.80; Z = 18.09; *p* < 0.001). The single study using a machine learning method (n = 1) precluded assessment of heterogeneity. The limited number of machine learning–based studies highlights the need for larger, more diverse datasets to enable robust evaluation and validation of such models.

Subgroup analyses indicated that the effect size of the readmission prediction models was significantly influenced by publication year, geographic region, and type of readmission outcome. The observed heterogeneity appeared to arise primarily from differences in outcome definitions (e.g., MACE) and methodological variability (e.g., modeling approaches). To improve comparability and generalizability, future studies should aim to standardize outcome definitions and strengthen the external validation of machine learning–based models. Egger’s test (*p* = 0.06) indicated no significant evidence of publication bias (see **Supplementary Fig. 1**).

## 4. Discussion

### 4.1 Model Performance and Quality Analysis of Study

We evaluated 10 risk prediction models, of which all but the study by Minges *et al*. [37] showing moderate to good predictive performance during internal or external validation. Reported AUC values ranged between 0.660 and 0.899. However, based on the PROBAST checklist, all studies were classified as having a high risk of bias, which limits the generalizability of these risk prediction models. The pooled AUC for the nine models included in the meta-analysis was 0.80 (95% CI: 0.74–0.85). Pooling AUC estimates across studies enables a quantitative synthesis of the discriminative performance of readmission prediction models following PCI. Although AUC is a widely used and robust metric for assessing a model’s ability to rank patient risk, it is sensitive to outcome prevalence and calibration characteristics [41]. Thus, while meta-analysis of AUCs facilitates comparison of model performance across heterogeneous settings, it should not be interpreted as a direct indicator of model generalizability. To account for potential biases arising from differences in model specifications—such as variations in predictor sets or outcome timeframes—subgroup analyses were conducted based on publication year, study region, readmission type, and modeling approach. High heterogeneity across the studies may be attributed to differences in outcome definitions or methodological approaches.

Despite variability in performance and quality, these models provide valuable insights for future research. For instance, the study by Minges *et al*. [37], which employed a large-sample prospective design, relied on random split validation for internal validation. Although this method is a type of internal validation, it does not account for issues such as model overfitting [42], rendering it less favorable. The study by Zhang *et al*. [38] faced challenges such as a sample size yielding an EPV ratio below 20 and data from a single-center retrospective design in northwest China, leading to risks of bias in the participants, predictors, and outcome domains. Nevertheless, the study excelled in the analysis domain by using multiple imputation for missing data (<10%) and employing three robust predictor selection methods: LASSO regression, random forest, and best subset selection. Some models overlooked the importance of avoiding univariate analysis during predictor screening, a limitation that should be addressed in future studies.

The study by Liu *et al*. [32] integrated traditional logistic regression with machine learning methods during model development. Evidence suggests that, compared to traditional approaches, machine learning offers superior nonlinear fitting capabilities, enabling the capture of complex relationships and improving both predictive accuracy and model robustness [43]. Machine learning also demonstrates advantages in handling large-scale, high-dimensional, and incomplete datasets, and supports continuous model updating as new data become available—enhancing adaptability and long-term performance. However, machine learning methods are not without limitations. They are prone to overfitting, particularly when applied to small datasets; often lack transparency, reducing interpretability relative to traditional statistical models; and typically require large volumes of high-quality, complete data to function optimally. Moreover, the “black box” nature of some advanced algorithms may hinder their clinical applicability in contexts where interpretability is essential. Traditional methods such as logistic regression remain valuable, especially in scenarios involving smaller datasets or when clinical interpretability is prioritized. Ultimately, model selection should be guided by the specific characteristics of the research question, including data size and quality, the complexity of the associations involved, and the trade-off between interpretability and predictive performance.

Recent advances in machine learning have substantially improved risk prediction models in cardiovascular medicine. For example, Yilmaz *et al*. [44], demonstrated that machine learning algorithms leveraging electrocardiogram (ECG) features—such as P-wave, QRS complex (the combination of Q, R, and S waves in the electrocardiogram representing ventricular depolarization), and T-wave characteristics—can accurately predict obstructive coronary artery disease in patients undergoing high-risk treadmill exercise testing. Similarly, Cicek *et al*. [45] highlighted the potential of deep learning models for predicting short-term mortality in patients with acute pulmonary embolism. These studies illustrate a paradigm shift toward more sophisticated, data-driven approaches in cardiovascular risk prediction. While the current review primarily focused on conventional statistical models, future research should increasingly incorporate advanced machine learning techniques to enhance the accuracy of post-PCI readmission prediction. Standardized machine learning frameworks that integrate multimodal data—including clinical, imaging, and electrophysiological variables—may outperform traditional risk scores and offer greater predictive utility in diverse clinical settings.

Overall, while the included models exhibited moderate to good performance, the high risk of bias underscores the need for improvements. Future research should focus on optimizing sample sizes, addressing missing data, refining predictor selection, accounting for data complexity, and improving model fitting.

### 4.2 The Predictors Used in Prediction Model

The frequently identified predictors carry significant implications for nursing practice and future research.

Elderly patients are at heightened risk of hospital readmission due to age-related declines in cognitive and organ function, diminished self-care capacity, and a higher burden of comorbidities [46, 47]. These risk factors are comprehensively captured by the Intermountain Risk Score (IMRS), which integrates age, sex, and routine laboratory markers to predict post-PCI outcomes. IMRS has demonstrated strong prognostic value for both short- and long-term mortality in patients with ST-segment elevation myocardial infarction (STEMI) and cardiogenic shock, with advanced age being a heavily weighted component [48, 49]. These findings highlight the importance of targeted management strategies for elderly patients, including enhanced postoperative monitoring and individualized rehabilitation programs.

Female sex is independently associated with higher readmission rates following STEMI, a disparity that is reflected in IMRS-based risk stratification. While biological factors—such as heightened pain sensitivity and a greater comorbidity burden—partially explain this difference [50, 51, 52, 53, 54, 55, 56, 57], the IMRS further quantifies sex-specific risk, facilitating early identification of high-risk female patients [48]. Given the validated prognostic utility of IMRS in predicting post-discharge adverse events [49], structured interventions—including motivational follow-up and psychological support—should be prioritized for this subgroup to improve outcomes.

The bidirectional relationship between diabetes and coronary heart disease (CHD) complicates the postoperative recovery of patients with coexisting conditions. Stress-related hyperglycemia during angina or myocardial infarction, combined with vascular endothelial damage from high glucose levels, exacerbates CHD [58]. Consequently, patients with diabetes face heightened readmission risks due to challenges in disease management. Clinical practices should emphasize frequent follow-ups, strict blood glucose control, and targeted interventions to slow plaque progression, thereby reducing recurrent acute coronary syndrome (ACS) and readmission rates. A comprehensive approach, considering the interplay of diabetes, cardiovascular disease, and aging can lead to better treatment outcomes and enhanced patient quality of life.

Impaired cardiac output in heart failure patients increases their vulnerability to adverse cardiovascular events following PCI, elevating the risk of readmission. Interventions should focus on managing heart failure symptoms and optimizing post-PCI care to minimize complications.

The coexistence of chronic lung disease and cardiovascular disease intensifies readmission risks due to shared inflammatory pathways and oxygen supply-demand imbalances. Studies indicate that integrated prevention strategies enhance treatment efficacy [59]. Mechanisms such as inflammatory activation leading to plaque instability and myocardial hypoxia further exacerbate cardiovascular risk [60]. Observational data show a 3.8-fold increase in adverse cardiovascular events within 30 days after acute exacerbations of chronic obstructive pulmonary disease (COPD) [61]. For PCI patients with concurrent lung disease, integrating lung rehabilitation into care plans is crucial.

Patients with multivessel disease face higher risks of MACE due to extensive myocardial ischemia [62]. Studies associate this condition with severe atherosclerosis, poorly managed comorbidities, and compromised cardiac function [63]. A staged PCI approach, treating the culprit vessel first and addressing remaining lesions later, is recommended to improve outcomes [64].

The clinical implementation of readmission prediction models for post-PCI patients remains critical. However, further clinical trials are necessary to validate their efficacy in reducing readmission rates and expanding their applicability to broader patient populations. Such efforts would enhance the utility of these models in optimizing patient outcomes.

## 5. Limitations

Several limitations should be acknowledged in this study. First, some prediction models have not undergone external validation, leaving their generalizability unverified. Second, the exclusion of grey literature—such as trial registries and unpublished data—may have introduced publication bias into our analysis. As a result, the findings may not fully reflect the entire body of evidence on PCI readmission risk prediction models, particularly studies reporting negative or null results that are less likely to appear in peer-reviewed literature. Third, despite conducting subgroup analyses based on region, readmission type, and modeling methods, substantial residual heterogeneity persisted (I^2^ = 97%). This is likely attributable to unmeasured variability across studies, including differences in PCI protocols, post-discharge care practices, and patient-level confounders such as socioeconomic status. Additionally, inconsistent definitions of key outcomes—such as “MACE”—further contributed to heterogeneity and limited cross-study comparability. Fourth, certain predictive factors reported in only a single study could not be meta-analyzed, limiting their interpretability. Fifth, the limited number of studies within key subgroups—such as those employing machine learning models (n = 1)—restricted the ability to conduct more detailed analyses of methodological heterogeneity. Finally, language restrictions may have introduced selection bias, as only English and Chinese publications were included, potentially omitting relevant data from other major languages. Future research should prioritize individual participant data (IPD) meta-analyses to better address heterogeneity and facilitate model validation across diverse populations. Additionally, the inclusion of grey literature should be actively pursued to mitigate the risk of publication bias.

## 6. Conclusion

This systematic review included 10 studies reporting 18 prediction models for hospital readmission after PCI. The results indicated that the combined AUC for the nine validated models was 0.80 (95% confidence interval: 0.74–0.85), reflecting a moderate level of discriminatory ability. However, all included studies were evaluated as having a high risk of bias based on the PROBAST checklist, and six studies raised concerns about their applicability. At present, the prediction models for readmission after PCI do not meet PROBAST standards.

To improve the quality of future research, it is imperative for researchers to become well-versed in the PROBAST checklist and adhere to the reporting guidelines set forth in the Transparent Reporting of a Multivariable Prediction Model for Individual Prognosis or Diagnosis (TRIPOD) statement. Future studies should prioritize developing robust prediction models with larger sample sizes, more rigorous methodological designs, and multi-center external validation.

## Availability of Data and Materials

This study is a systematic review and meta-analysis based on published literature; no original datasets were generated. The data underlying this article are available in the article and in its online **Supplementary Material**.

## Supplementary Material

Supplementary material associated with this article can be found, in the online version, at https://doi.org/10.31083/RCM39409.
