# Supplementary material for: Risk Prediction Models for Hospital Readmission After Percutaneous Coronary Intervention: A Systematic Review and Meta-Analysis
Source: Rev Cardiovasc Med. 2025 Sep 12;26(9):39409. doi: 10.31083/RCM39409 (PMC12516748; doi:10.31083/RCM39409)
Supplement: Supplementary file 1 [file 2153-8174-26-9-39409-s1.docx]

**Supplementary Material**

**List of Supplementary Material**

- Supplementary Table 1. PICOTS
- Supplementary Table 2. Complete List of Search Terms
- Supplementary Table 3. CHARMS checklist
- Supplementary Table 4. Characteristics of PCI readmission risk prediction models
- Supplementary Table 5. Risk of bias assessment tables and key for risk of bias assessment
- Supplementary Table 6. PRISMA Checklist
- Supplementary Fig. 1. Funnel plot

**Supplementary Table 1. PICOTS**

| Item | Criteria |
| --- | --- |
| Population | Patients after PCI for coronary heart disease |
| Index prediction model | Risk prediction models for readmission after PCI that were developed and published (predictors ≥ 2) |
| Comparator | No competing model |
| Outcome | The occurrence of readmission post-PCI |
| Timing | The outcome was predicted after evaluating basic information at admission, cardiac status, and laboratory indicators |
| Setting | The intended use of the risk prediction is to individualize the prediction of readmission in patients undergoing PCI, facilitating the implementation of preventive measures to prevent adverse events |

**Supplementary Table 2. Complete List of Search Terms**

| Search Terms |
| --- |
| (patient readmission[MESH] OR “readmission, patient”[Title/Abstract] OR “unplanned readmission*”[Title/Abstract] OR rehospitalization*[Title/Abstract] OR “hospital readmission*”[Title/Abstract])  AND  (“percutaneous coronary intervention”[MESH] OR “percutaneous coronary intervention”[Title/Abstract] OR “percutaneous coronary revascularization*”[Title/Abstract] OR “coronary intervention*, percutaneous”[Title/Abstract] OR “coronary revascularization, percutaneous”[Title/Abstract] OR PCI[Title/Abstract])  AND  (“risk assessment”[MESH] OR “risk assessment”[Title/Abstract] OR predict*[Title/Abstract] OR “predictive model”[Title/Abstract] OR “predicting model”[Title/Abstract] OR “risk score”[Title/Abstract] OR “risk model”[Title/Abstract] OR “prognostic model”[Title/Abstract]) OR “risk prediction”[Title/Abstract] OR “risk factors*”[Title/Abstract] |

MESH, Medical Subject Headings; PCI, percutaneous coronary intervention

**Supplementary Table 3. CHARMS 2014 checklist**

| Domain | Key entries |
| --- | --- |
| 1 Data sources | The data sources included various study designs such as cohort studies, case-control studies, randomized controlled trials (RCTs), and registry studies. |
| 2 Study Population | 2.1 Study Population Criteria and Recruitment Methods (e.g., consecutive sampling, locations, number of study centers, setting, inclusion and exclusion criteria) |
|  | 2.2 Characteristics of Study Participants |
|  | 2.3 Detailed Description of Treatment Received (if applicable) |
|  | 2.4 Study Dates |
| 3 Outcomes to be predicted | 3.1 Definition and measurement method of outcomes |
|  | 3.2 Was the same outcome definition (and measurement method) used for all patients? |
|  | 3.3 Type of outcome |
|  | 3.4 Were outcome assessors blinded to candidate predictors? |
|  | 3.5 Are candidate predictors part of the outcome? |
|  | 3.6 Total duration of outcome occurrence or follow-up time |
| 4 Candidate Predictors | 4.1 Number and types of predictors (e.g., demographic characteristics, medical history, physical examination findings, other test results, disease features) |
|  | 4.2 Definition and measurement methods of candidate predictors |
|  | 4.3 Timing of predictor measurement |
|  | 4.4 Were predictors assessed without knowledge of outcomes? (i.e., blinded assessment) |
|  | 4.5 Handling of predictors (e.g., continuous, linear, nonlinear transformation, or categorical) |
| 5 Sample Size | 5.1 Study sample size and number of outcomes/events |
|  | 5.2 Number of outcomes/events relative to candidate predictor variables (events per variable) |
| 6 Missing Data | 6.1 Number of study samples with any missing values in predictors or outcomes |
|  | 6.2 Number of study samples with missing data for each predictor |
|  | 6.3 Handling of missing data (e.g., complete case analysis, imputation, or other methods) |
| 7 Model Development | 7.1 Modeling Approach (e.g., logistic regression, survival analysis, neural networks, or machine learning techniques) |
|  | 7.2 Compliance with Modeling Assumptions |
|  | 7.3 Method for Selecting Predictors for Inclusion in Multivariable Models (e.g., all candidate predictors, preselection based on unadjusted associations with the outcome) |
|  | 7.4 Method for Selecting Predictors During Multivariable Modeling (e.g., full model approach, backward or forward selection) and Criteria Used (e.g., p-value, etc.) |
|  | 7.5 Method for Shrinkage of Predictor Weights or Regression Coefficients (e.g., no shrinkage, uniform shrinkage, penalized estimation) |
| 8 Model Performance | 8.1 Calibration (calibration plot, calibration slope, goodness-of-fit test) and discrimination (C-statistic, D-statistic, time-dependent ROC analysis) with their confidence intervals |
|  | 8.2 Classification metrics (including: sensitivity, specificity, predictive values, net reclassification improvement) and whether a priori cutoff points were used |
| 9 Model Evaluation | 9.1 Methods used to test model performance: Development dataset only (random split of data, resampling methods, e.g., bootstrap, cross-validation, or none) or separate external validation (e.g., temporal, geographical, different settings, different investigators) |
|  | 9.2 Whether the model was adjusted or updated when validation performance was unsatisfactory (e.g., recalibration of intercept, adjustment of predictor effects, or addition of new predictors) |
| 10 Results | 10.1 Final model and other multivariable models (e.g., basic model, extended model, reduced model), including predictor weights or regression coefficients, intercept, baseline survival rate, and model performance measures (with their standard errors or confidence intervals). |
|  | 10.2 Any presentation formats of the final prediction model (e.g., summary scores, nomogram, scoring table, predictive performance in specific risk subgroups). |
|  | 10.3 Comparison of predictor distributions (including missing data) between development and validation datasets. |
| 11 Interpretation and Discussion | 11.1 Explanation of the Proposed Model (Validation results: Does the model demonstrate practical utility? Or is it exploratory in nature, requiring further investigation?) |
|  | 11.2 Comparative Analysis (Discussion of generalizability, advantages, and limitations in comparison with existing studies.) |

**Supplementary Table 4. Characteristics of PCI readmission risk prediction models**

| **Author (year)** | **Missing data handling** | **Continuous variable processing method** | **Variable selection** | **Model development method** | **Calibration method** | **Validation method** | **Final predictors** | **Model performance** | **Model presentation** |
| --- | --- | --- | --- | --- | --- | --- | --- | --- | --- |
| Wasfy 2013 | Creating a category for missing data and conducting a sensitivity analysis to compare the results before and after including the independent category | Continuous variables | Backwards elimination | - | Hosmer-Lemeshow test | Split random (66% developmental cohort and 34% validation cohort) | female sex, previous CABG, current CHF, chronic lung disease, peripheral arterial disease, cardiogenic shock, age, GFR, admission status, insurance status | A:  Model 1: 0.680  Model 2: 0.690  B: 0.670 | Risk score, web-based calculator |
| Minges 2017 | - | Categorical variables | Stepwise selection(logistic regression model) | - | - | Split random (50% developmental cohort and 50% validation cohort) | age, female, BMI, history of heart failure, previous valvular surgery, peripheral vascular disease, chronic lung disease, diabetes, GFR, renal failure - dialysis, current heart failure, symptoms on admission, EF percentage, PCI status | A: 0.670  B: 0.660 | Risk score |
| Fanari 2017 | - | Continuous variables | combination of forward selection and backward elimination | logistic regression model | Calibration plot | Bootstrapping & temporal validation | **Risk factors:** increasing age, female gender, previous hospitalization within 6 months of the current hospitalization, total count of Elixhauser comorbidities, CHF, renal failure, depression, discharge to SNF, Canadian Classification System angina class  **Protective factors:** private insurance carrier, elective procedure | A: 0.752  (0.724,0.781) | - |
| Zack 2019 | PCI events with more than 50% of data missing were excluded; remaining missing values were imputed with mean or mode of the data | Continuous variables | - | logistic regression model, RF | - | Cross-validation | length of stay, antiarrhythmic medication, inotrope use | Model 1: 0.899  (0.890,0.910)  Model 2: 0.846  (0.840,0.860) | - |
| Xu 2022 | delete cases with more than 5% missing variables; fill in the remaining missing values with mean of the data | Categorical variables | Univariate analysis and LASSO regression | logistic regression model | Hosmer-Lemeshow test | Bootstrapping | Medical insurance, length of stay, left ventricular ejection fraction on admission, history of hypertension, the presence of chronic lung disease, the presence of anemia, serum creatinine level on admission | Model 1:  A:0.770  (0.746,0.792)Model 2:  0.734  (0.687,0.781) | Nomogram model |
| Zhang 2022 | - | Continuous variables | Univariate analysis | logistic regression model | Hosmer-Lemeshow test | - | age, diatebes, total cholesterol, triglyceride | A: 0.843 | Nomogram model |
| Zhang 2022 | - | Continuous variables | Univariate analysis | logistic regression model | - | External temporal validation | age(≥70 years old), hypertension, hyperlipidemia, diatebes mellitus, cerebrovascular disease, left heart failure, triple vessel lesion, left main coronary arterial lesion, stent number ≥ 3 | A: 0.828  B: 0.805 | Nomogram model |
| Yao 2022 | - | Continuous variables | Univariate analysis; clinical relevance, and the number of events available | logistic regression model | Hosmer-Lemeshow test | Internal validation | female gender, hypertension, in-hospital VA   1. vessel disease, baseline NT-proBNP, peak cTnI | A: 0.723  (0.665,0.780) | Nomogram model |
| Zhang 2024 | Delete variables with more than 20% missing data, and delete cases with more than 10% missing variables; fill in the remaining missing values using multiple imputation | Continuous variables | LASSO regression; RF; best subset selection | logistic regression model | - | Bootstrapping | age, hospitalization days, diabetes history exceeding 20 years, cystatin C, ejection fraction, multivessel disease, ST/T wave changes | A: 0.787  (0.760,0.815) | Nomogram model |
| Liu 2024 | - | Continuous variables | Univariate and multivariable logistic regression model; LASSO regression; RF | logistic regression model, DT, RF,  SVM, XGBoost, AdaBoost | Calibration curve | Internal temporal validation | discharge outcomes, admission mode (walking and non-walking), communication ability, CRP, TC, HDL, LDL | Model 1: 0.749  (0.681,0.817)  Model 2: 0.665  (0.597,0.733)  Model 3: 0.702  (0.628,0.776)  Model 4: 0.728  (0.660,0.795)  Model 5: 0.729  (0.661,0.798)  Model 6: 0.706  (0.633,0.779) | - |
| **Abbreviations:** PCI, percutaneous coronary intervention; LASSO, least absolute shrinkage and selection operator regression; RF, Random Forest; DT, Decision Tree; SVM, support vector machine; XGBoost, extreme gradient boosting; AdaBoost, Adaptive Boosting; CABG, coronary artery bypass graft; CHF, congestive heart failure; GFR, glomerular filtration rate; BMI, body mass index; EF, ejection fraction; CHF, chronic heart failure; SNF, skilled nursing facility; VA, Ventricular arrhythmias; NT-proBNP, N-terminal pro-B-type natriuretic peptide; cTnI, Cardiac Troponin I; CRP, C-reactive protein; TC, Total Cholesterol; HDL, high-density lipoprotein; LDL, low-density lipoprotein.  A, development cohort; B, validation cohort.  AUC, area under the curve. We considered AUC = 0.5-0.7 as poor discrimination, 0.7-0.8 as moderate discrimination, 0.8-0.9 as good discrimination, and 0.9-1.0 as excellent discrimination. | | | | | | | | | |

**Supplementary Table 5.** **Risk of bias assessment tables and key for risk of bias assessment**

**Risk of bias assessment: population, predictors and outcomes (based on PROBAST)**

| **Study** | **Model** | **Appropriate data source?** | **In/exclusions of participants appropriate?** | **Predictors defined and assessed in similar way for all participants?** | **Predictor assessment made without knowledge of outcome data?** | **All predictors available at the time the model is intended to be used?** | **Outcome determined appropriately?** | **Pre-defined/standard outcome definition?** | **Predictors excluded from the outcome definition?** | **Outcome defined and determined in a similar way for all participants?** | **Outcome determined without knowledge of predictor information?** | **Appropriate time interval between predictor and outcome assessment?** |
| --- | --- | --- | --- | --- | --- | --- | --- | --- | --- | --- | --- | --- |
| Wasfy et al. | **DEV √**  **INT VAL √**  EXT VAL X | PY | We excluded PCIs in patients who could not be matched between the 2 data sets (n=2726), were not residents of Massachusetts (n=2998), had incomplete data (n=57), or did not survive the index hospitalization (n=589) | PY | PY | Y* | Y | Y | Y | PY | NI | Y |
| Minges 2017 | **DEV √**  **INT VAL √**  EXT VAL X | Y | For the current study we applied the same exclusions as for the readmission measure: admitted to the hospital less than 65 years of age; same-day discharges (patients would not be captured in the Part A inpatient data); admissions to hospitals with a missing, invalid, or duplicate Medicare Provider Number (MPN), and admissions in which the patients had identical information regarding age, sex, admission date, discharge date, and hospital MRN;  patients admitted to the hospital who died during the index hospitalization or were transferred to another acute care facility and did not receive a PCI; admissions for patients with incomplete administrative data for the period 12 months prior to the index admission date or for the 30 days following discharge from the index hospitalization; procedures that were not the first PCI in hospital stay; PCI admissions within 30 days of discharge from an index PCI admission; patients who underwent staged PCI; patients who left the hospital against medical advice; patients not enrolled in Medicare Part A (fee-for-service); and when the PCI was performed >10 days following admission to the hospital. We also excluded patients who were missing data on the type of PCI (elective, urgent, emergency, salvage). | PY | PY | Y | Y | Y | Y | PY | PY | Y |
| Fanari 2017 | **DEV √**  **INT VAL √**  **EXT VAL √** | PY | We excluded elective readmissions such as staged PCIs based on admission records. We excluded patients who were who received coronary artery bypass surgery during the same admission. | PY | PY | Y* | Y | Y | Y | PY | NI | Y |
| Zack 2019 | **DEV √**  **INT VAL √**  EXT VAL X | Y | Patients who electively declined authorization for use of their medical records in research and those with health  care paid for by the Federal Bureau of Prisons or by non-U.S. governments were excluded. | PY | PY | Y | Y | Y | Y | PY | PY | Y |
| Xu 2022 | **DEV √**  **INT VAL √**  EXT VAL X | PY | The exclusion criteria were as follows: (i) Patients with end-stage liver disease, end-stage renal disease, or a diagnosis  of malignancy; (ii) patients with known severe mental illness or cognitive dysfunction; and (iii) patients with incomplete  clinical or laboratory data. | PY | PY | Y* | Y | Y | Y | PY | NI | Y |
| Zhang M 2022 | **DEV √**  INT VAL X  EXT VAL X | PY | Exclusion Criteria:  (1) comorbid malignancies or severe organic brain diseases;  (2) presence of poorly controlled chronic conditions, including renal disease, pulmonary complications, hypertension, and diabetes mellitus; (3) patients with cognitive impairment. | PY | PY | Y | PY | PY | Y | PY | NI | Y |
| Zhang L 2022 | **DEV √**  **INT VAL √**  **EXT VAL √** | PY | Exclusion criteria were as follows: recent history of surgical intervention; severe impairment of cardiac, pulmonary, hepatic or renal function; diagnosis of malignancy; incomplete clinical data or lost to follow-up. | PY | PY | Y* | PY | NI | Y | PY | NI | Y |
| Yao 2022 | **DEV √**  **INT VAL √**  EXT VAL X | PY | The exclusion criteria included  (1) in-hospital death, (2) more than 10% data missing, and (3)  readmission to the hospital for non-MACEs. | PY | PY | Y* | PY | Y | Y | PY | NI | Y |
| Zhang 2024 | **DEV √**  **INT VAL √**  EXT VAL X | PY | The exclusion criteria were as follows: (1) presence of other relevant clinical disorders (e.g., hepatic or renal insufficiency, severe infections, autoimmune deficiency, malignant tumors, or advanced neurological diseases); (2) presence of cognitive impairment or psychiatric disorders; and (3) missing clinical medical records. | PY | PY | Y* | NI | NI | Y | PY | NI | Y |
| Liu 2024 | **DEV √**  **INT VAL √**  EXT VAL X | PY | 213 patients were excluded (47 died during hospitalization, and 166 had incomplete data). | PY | PY | Y* | Y | Y | Y | PY | NI | Y |

AF = atrial fibrillation; CABG = coronary artery bypass graft; CAD = coronary artery disease; COMP=study which compares two or more models; DEV=model development study; EDW = Enterprise Data Warehouse; EXT VAL =study with external validation of a model; INT VAL= study with internal model validation; LVEF=left ventricular ejection fraction; N=no; NEI=not enough information; NI=no information; PCI = Percutaneous Coronary Intervention; PN=probably no; PY=probably yes; U/M =study which updates or modifies a model; Y=yes.

* relates to discharge to SNF, length of stay, triple vessel lesion, left main coronary arterial lesion, stent number ≥ 3, discharge outcomes, as a variable for prediction of readmission, so variables cannot be used pre-procedurally

**Risk of bias assessment: analysis (based on PROBAST)**

| **Study** | **Model** | **Was there a reasonable number of participants with the outcome?** | **Were continuous and categorical predictors handled appropriately? For validation: was model evaluated as originally fitted?** | **Were all enrolled participants included in the analysis?** | **Were participants with missing data handled appropriately?** | **Was selection of predictors based on univariate analysis avoided? (DEV only)** | **Were complexities in the data (e.g. censoring, competing risks, sampling of control participants) accounted for appropriately?*** | **Were relevant model performance measures evaluated appropriately?** | **Were model overfitting and optimism in model performance accounted for? (DEV only)** | **Do predictors and their assigned weights in the final model correspond to the results from the reported multivariable analysis? (DEV only)** |
| --- | --- | --- | --- | --- | --- | --- | --- | --- | --- | --- |
| Wasfy et al. | **DEV √**  **INT VAL √**  EXT VAL X | Y  34 candidate variables; 2500 events. 74 EPV. | Y | Y  All consecutive patients  undergoing PCI included for analysis. | PY | Y  Multivariate analysis to identify variables | NI | Y  (NB assessment of calibration based on Hosmer-Lemeshow test which has limited power). | PN  Split random | NI |
| Minges 2017 | **DEV √**  **INT VAL √**  EXT VAL X | Y  20 candidate variables; 44066 events. 2203 EPV. | PN | Y  All consecutive patients  undergoing PCI included for analysis. | Y | Y  Multivariate analysis to identify variables | NI | N  No calibration measures reported. | PN  Split random | NI |
| Fanari 2017 | **DEV √**  **INT VAL √**  **EXT VAL √** | PY  27 candidate variables; 318 events. 12 EPV. | Y | Y  All consecutive patients  undergoing PCI included for analysis. | NI | Y  Multivariate analysis to identify variables | NI | Y | Y  Bootstrapping | NI |
|  |  | PY for EXT VAL  2978 patients. | Y |  |  |  | NI |  |  |  |
| Zack 2019 | **DEV √**  **INT VAL √**  EXT VAL X | N  410 candidate variables; 82 events. 0.2 EPV. | NI | NI | PY | NI | NI | N  No calibration measures reported. | Y  Cross-validation | NI |
| Xu 2022 | **DEV √**  **INT VAL √**  EXT VAL X | N  33 candidate variables; 107 events. 3 EPV. | Y | NI | PY | Y  LASSO method to identify variables | NI | Y  (NB assessment of calibration based on Hosmer-Lemeshow test which has limited power). | Y  Bootstrapping | Y |
| Zhang M 2022 | **DEV √**  INT VAL X  EXT VAL X | N  29 candidate variables; 42 events. 1 EPV. | Y | Y  All consecutive patients  undergoing PCI included for analysis. | NI | N  Included variables identified as significant on univariate analysis. | NI | Y  (NB assessment of calibration based on Hosmer-Lemeshow test which has limited power). | N  No internal validation. | Y |
| Zhang L 2022 | **DEV √**  INT VAL X  **EXT VAL √** | N  18 candidate variables; 133 events. 7 EPV. | Y | Y  All consecutive patients  undergoing PCI included for analysis. | NI | N  Included variables identified as significant on univariate analysis. | NI | N  No calibration measures reported. | NI | Y |
|  |  | Y for EXT VAL  108 events. | Y |  |  |  | NI |  |  |  |
| Yao 2022 | **DEV √**  **INT VAL √**  EXT VAL X | N  36 candidate variables; 70 events. 2 EPV. | Y | Y  All consecutive patients  undergoing PCI included for analysis. | NI | N  Included variables identified as significant on univariate analysis. | NI | Y  (NB assessment of calibration based on Hosmer-Lemeshow test which has limited power). | NI | Y |
| Zhang 2024 | **DEV √**  **INT VAL √**  EXT VAL X | PY  22 candidate variables; 358 events. 16 EPV. | Y | NEI  Patients with incomplete clinical data excluded | Y | Y  LASSO method to identify variables | NI | N  No calibration measures reported. | Y  Bootstrapping | Y |
| Liu 2024 | **DEV √**  **INT VAL √**  EXT VAL X | N  96 candidate variables; 226 events. 2 EPV. | Y | Y  All consecutive patients  undergoing PCI included for analysis. | NI | Y  LASSO, LR and RF methods to identify variables | NI | Y | PY  Temporal validation | NI |

COMP=study which compares two or more models; DEV=model development study; EPV=events per variable; EXT VAL =study with external validation of a model; INT VAL= study with internal model validation; N=no; NEI=not enough information; NI=no information; PN=probably no; PY=probably yes; U/M =study which updates or modifies a model; Y=yes.

**Risk of bias assessment –key (informed by PROBAST)**

| *Appropriate data source?* | Y if prospective cohort with consecutive PCI patients or all patients within a specified timeframe.  PY if prospective cohort (limited details) or retrospective analysis with consecutive patients (or all PCI patients admitted during a defined period).  NEI if retrospective analysis without details or if single-center study with insufficient information.  PN if data based on a sub-group from a larger PCI cohort. |
| --- | --- |
| *Were all inclusions and exclusion appropriate?* | No decision made on whether these were appropriate due to (i) The inconsistency in readmission types (e.g., all-cause readmission, cardiovascular-related readmission, or MACE-associated readmission). Reported details are tabulated where available. |
| *Predictors defined and assessed in similar way for all participants?* | Y if reference made to standard criteria used in all patients.  PY if single centre. For some criteria there is a standard way of measuring (e.g. LVEF), others are not prone to measurement issues (e.g. age, sex). Some information is unlikely to be reported, e.g. how co-morbidities were defined.  PY if states that methods performed in accordance with guidelines for cardiac surgery or atrial fibrillation management.  PY if multicentre but standardised protocol.  NI if not able to tell if single centre and no other information.  N if specific statement that variables were measured in different ways. |
| *Predictor assessment made without knowledge of outcome data?* | Y if clear that all score components measured before PCI (and statement to that effect).  PY if appears that (at least some) predictors were measured pre-procedurally (or at admission). There are some standard pre-procedural investigations. Less important for fixed predictors (age, sex).  NI if no details on timing of predictor assessment. |
| *All predictors available at the time the model is intended to be used?* | This is always Y, except models incorporation postoperative early predictors (eg., postoperative stroke) which are unknown before surgery. This has been indicated in the table. |
| *Was the outcome determined appropriately?* | Y if readmission was ascertained through comprehensive methods (e.g., hospital records, claims data, or active follow-up with standardized criteria).  PY if readmission was mentioned but methods were unclear or relied partially on patient self-report without validation.  N If readmission was determined solely through unverified sources (e.g., unstructured chart review without clear criteria).  NI if no definition was provided. |
| *Standard outcome definition?* | Y if readmission was clearly defined (e.g., unplanned hospitalization within 30 days post-PCI, excluding planned readmissions like staged procedures).  N if readmission was described but without clear exclusions (e.g., "any hospitalization" without distinguishing planned vs. unplanned).  NI if no definition was provided. |
| *Were predictors excluded from the outcome definition?* | This is always Y. Predictors do not form part of outcome assessment. |
| *Was the outcome defined and determined in a similar way for all participants?* | Y if explicit statement.  PY if the standard definition given and/or single centre. |
| *Was the outcome determined without knowledge of predictor information?* | NI if study reported no information regarding this.  N if clear statement that outcome assessment could have been influenced by knowledge of one or more variables. |
| *Appropriate time interval between predictor and outcome assessment?* | Y - if follow-up covered ≥30 days post-PCI (capturing the high-risk period for readmission).  PY- if follow-up was likely adequate (e.g., median/mean reported but minimum unclear).  NI –no information on follow-up duration.  PN –if most patients lacked 30-day follow-up (e.g., data only during index hospitalization). |
| *Was there a reasonable number of participants with the outcome?* | Development studies  Y -if >20 events per variable for candidate predictors  PY –if > 10 events per variable  NI- no details or unclear how many candidate variables  PN- if number of candidate variables unclear but small sample size  N -<10 events per variable  Validation studies  Y-at least 100 participants with outcome.  PY –if events not stated but very large sample size (e.g. >1000)  NI-number of events not stated  N-less than 100 participants with outcome |
| *Were continuous and categorical predictors handled appropriately?* | Development studies  Y –if no dichotomisation of continuous predictors based on study data or if cut-off predefined (widely accepted) rather than based on the data  NI- no details  PN - appears that one or more cut-offs based on study data  N-if dichotomisation of one or more continuous predictors based on study data (and no adjustment by applying internal validation and shrinkage techniques)  Validation studies  Y- if model being used as originally fitted-same dichotomisation and cut-offs. Using equation/model as created.  PY- appears that same model is being used but not explicit  PN-appears that some model variables have been changed  N-some model variables (or cut-offs) clearly changed |
| *Were all enrolled participants included in the analysis?* | Y- explicit statement that all patients were included in analysis (or if some were excluded –that the characteristics were similar to the included)  NI-no details, or small proportion (<10%) of patients excluded but no information on similarity of patient characteristics between in- and excluded  N-clear statement that >10% patients lost to follow-up (or with missing predictor information) were excluded from the analysis and/or that patient characteristics differed between in-and excluded  Note that sometimes availability of variable or outcome data was as an eligibility criterion. |
| *Were participants with missing data handled appropriately?* | 1. an explicit statement that all enrolled patients were included in the final analysis or studies reported an appropriate method for handling missing data, e.g. by using multiple imputation.   PY- if excluded had similar characteristics to included  NI- no details on handling of missing data  N- patients lost to follow-up simply excluded (and no details on similarity of patients characteristics between in- and excluded) |
| *Was selection of predictors based on univariate analysis avoided? (DEV only)* | Y- if predictors identified through multivariate analysis  NI –no details on how predictors were selected  N- if predictors selected on the basis of univariable analysis before multivariable modelling |
| *Were complexities in the data (e.g. censoring, competing risks, sampling of control participants) accounted for appropriately?* | Development studies  Y- time to event analysis used (e.g. Cox analysis)  NI-no details on type of analysis  PN- logistic regression model used/insufficient information  N-no time-to event analysis used  Validation studies  N/A as none of the validation studies undertook calibration and model refitting. |
| *Were relevant model performance measures evaluated appropriately?* | Y-both a discrimination and calibration statistic reported  N-only one of the above or none presented |
| *Were model overfitting and optimism in model performance accounted for? (DEV only)* | Y-a form of internal validation included (e.g. bootstrapping or cross-validation); where included should adjust or shrink the model predictive performance estimates and predictor effects in the final model  PN- a split sample approach used with >20 events per candidate variable  N-no form of internal validation or a split sample approach with <20 events per candidate variable |
| *Do predictors and their assigned weights in the final model correspond to the results from the reported multivariable analysis? (DEV only)* | Y-regression co-efficients used to estimate contribution of each variable to the risk  PY- score scale based on regression co-efficients (with no further details)  NI-no information on how weights assigned  N-inappropriate method, e.g. assigning points based on relative risk or degree of separation of Kaplan-Meier curves |

DEV=model development study; N=no; NEI=not enough information; NI=no information; PN=probably no; PY=probably yes; Y=yes.

**Supplementary Table 6. PRISMA Checklist**

|  | **Item #** | **Checklist item** | **Location where item is reported** |
| --- | --- | --- | --- |
| **TITLE** | | |  |
| Title | 1 | Identify the report as a systematic review. | 1 |
| **ABSTRACT** | | |  |
| Abstract | 2 | See the PRISMA 2020 for Abstracts checklist. | 1 |
| **INTRODUCTION** | | |  |
| Rationale | 3 | Describe the rationale for the review in the context of existing knowledge. | 1-2 |
| Objectives | 4 | Provide an explicit statement of the objective(s) or question(s) the review addresses. | 2 |
| **METHODS** | | |  |
| Eligibility criteria | 5 | Specify the inclusion and exclusion criteria for the review and how studies were grouped for the syntheses. | 2 |
| Information sources | 6 | Specify all databases, registers, websites, organisations, reference lists and other sources searched or consulted to identify studies. Specify the date when each source was last searched or consulted. | 2 |
| Search strategy | 7 | Present the full search strategies for all databases, registers and websites, including any filters and limits used. | 2 |
| Selection process | 8 | Specify the methods used to decide whether a study met the inclusion criteria of the review, including how many reviewers screened each record and each report retrieved, whether they worked independently, and if applicable, details of automation tools used in the process. | 2 |
| Data collection process | 9 | Specify the methods used to collect data from reports, including how many reviewers collected data from each report, whether they worked independently, any processes for obtaining or confirming data from study investigators, and if applicable, details of automation tools used in the process. | 2 |
| Data items | 10a | List and define all outcomes for which data were sought. Specify whether all results that were compatible with each outcome domain in each study were sought (e.g. for all measures, time points, analyses), and if not, the methods used to decide which results to collect. | 2 |
|  | 10b | List and define all other variables for which data were sought (e.g. participant and intervention characteristics, funding sources). Describe any assumptions made about any missing or unclear information. | 2 |
| Study risk of bias assessment | 11 | Specify the methods used to assess risk of bias in the included studies, including details of the tool(s) used, how many reviewers assessed each study and whether they worked independently, and if applicable, details of automation tools used in the process. | 2-3 |
| Effect measures | 12 | Specify for each outcome the effect measure(s) (e.g. risk ratio, mean difference) used in the synthesis or presentation of results. | 3-4 |
| Synthesis methods | 13a | Describe the processes used to decide which studies were eligible for each synthesis (e.g. tabulating the study intervention characteristics and comparing against the planned groups for each synthesis (item #5)). | 3-4 |
|  | 13b | Describe any methods required to prepare the data for presentation or synthesis, such as handling of missing summary statistics, or data conversions. | 3-4 |
|  | 13c | Describe any methods used to tabulate or visually display results of individual studies and syntheses. | 3-4 |
|  | 13d | Describe any methods used to synthesize results and provide a rationale for the choice(s). If meta-analysis was performed, describe the model(s), method(s) to identify the presence and extent of statistical heterogeneity, and software package(s) used. | 3-4 |
|  | 13e | Describe any methods used to explore possible causes of heterogeneity among study results (e.g. subgroup analysis, meta-regression). | 3-4 |
|  | 13f | Describe any sensitivity analyses conducted to assess robustness of the synthesized results. | 3-4 |
| Reporting bias assessment | 14 | Describe any methods used to assess risk of bias due to missing results in a synthesis (arising from reporting biases). | 2-3 |
| Certainty assessment | 15 | Describe any methods used to assess certainty (or confidence) in the body of evidence for an outcome. | 3-4 |
| **RESULTS** | | |  |
| Study selection | 16a | Describe the results of the search and selection process, from the number of records identified in the search to the number of studies included in the review, ideally using a flow diagram. | 4 |
|  | 16b | Cite studies that might appear to meet the inclusion criteria, but which were excluded, and explain why they were excluded. | 4 |
| Study characteristics | 17 | Cite each included study and present its characteristics. | 4 |
| Risk of bias in studies | 18 | Present assessments of risk of bias for each included study. | 4, 9 |
| Results of individual studies | 19 | For all outcomes, present, for each study: (a) summary statistics for each group (where appropriate) and (b) an effect estimate and its precision (e.g. confidence/credible interval), ideally using structured tables or plots. | 4-8 |
| Results of syntheses | 20a | For each synthesis, briefly summarise the characteristics and risk of bias among contributing studies. | 4-9 |
|  | 20b | Present results of all statistical syntheses conducted. If meta-analysis was done, present for each the summary estimate and its precision (e.g. confidence/credible interval) and measures of statistical heterogeneity. If comparing groups, describe the direction of the effect. | 9 |
|  | 20c | Present results of all investigations of possible causes of heterogeneity among study results. | 9-12 |
|  | 20d | Present results of all sensitivity analyses conducted to assess the robustness of the synthesized results. | 12 |
| Reporting biases | 21 | Present assessments of risk of bias due to missing results (arising from reporting biases) for each synthesis assessed. | NA |
| Certainty of evidence | 22 | Present assessments of certainty (or confidence) in the body of evidence for each outcome assessed. | NA |
| **DISCUSSION** | | |  |
| Discussion | 23a | Provide a general interpretation of the results in the context of other evidence. | 12-13 |
|  | 23b | Discuss any limitations of the evidence included in the review. | 14 |
|  | 23c | Discuss any limitations of the review processes used. | 14 |
|  | 23d | Discuss implications of the results for practice, policy, and future research. | 14 |
| **OTHER INFORMATION** | | |  |
| Registration and protocol | 24a | Provide registration information for the review, including register name and registration number, or state that the review was not registered. | 1, 2 |
|  | 24b | Indicate where the review protocol can be accessed, or state that a protocol was not prepared. | NA |
|  | 24c | Describe and explain any amendments to information provided at registration or in the protocol. | NA |
| Support | 25 | Describe sources of financial or non-financial support for the review, and the role of the funders or sponsors in the review. | 15 |
| Competing interests | 26 | Declare any competing interests of review authors. | 15 |
| Availability of data, code and other materials | 27 | Report which of the following are publicly available and where they can be found: template data collection forms; data extracted from included studies; data used for all analyses; analytic code; any other materials used in the review. | NA |


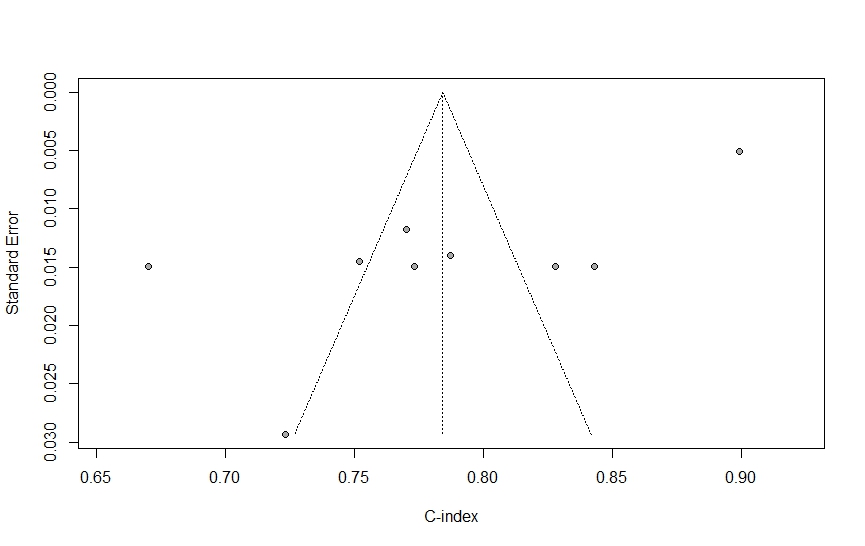


**Supplementary Fig. 1. Funnel plot**
